# Carbonic anhydrase modulation of emotional memory. Implications for the treatment of cognitive disorders

**DOI:** 10.1080/14756366.2020.1766455

**Published:** 2020-05-13

**Authors:** Patrizio Blandina, Gustavo Provensi, Maria Beatrice Passani, Clemente Capasso, Claudiu T. Supuran

**Affiliations:** a Department of Neurofarba, Section of Pharmacology and Toxicology, University of Florence, Firenze, Italy; b Department of Health Science, Section of Clinical Pharmacology and Oncology, University of Florence, Firenze, Italy; c Department of Biology, Agriculture and Food Sciences, CNR, Institute of Biosciences and Bioresources, Napoli, Italy; d Department of Neurofarba, University of Florence, Section of Pharmaceutical and Nutraceutical Sciences, Firenze, Italy

**Keywords:** Carbonic anhydrase, cognition-related disorders, CA isoforms, CA inhibitors, CA activators

## Abstract

Carbonic anhydrases (CAs, EC 4.2.1.1) are metalloenzymes which use CO_2_ as substrate, catalysing its interconversion to bicarbonate and a proton. In humans 15 CAs are expressed, 12 of which are catalytically active: the cytosolic CA I-III, VII, XIII, the membrane-bound CA IV, the mitochondrial CA VA and VB, the secreted CA VI, and the transmembrane CA IX, XII, XIV. Nine isoforms are present in the mammalian brain. Evidence supporting that CA inhibitors impair memory in humans has come from studies on topiramate and acetazolamide during acute high-altitude exposure. In contrast, administration of CA activators in animal models enhances memory and learning. Here we review the involvement of selective CA inhibition/activation in cognition-related disorders. CAs may represent a crucial family of new targets for improving cognition as well as in therapeutic areas, such as phobias, obsessive-compulsive disorder, generalised anxiety, and post-traumatic stress disorders, for which few efficient therapies are available.

## Memory and fear learning

Memory originates from experience and establishes the uniqueness of our personal history, as it affects the way we think, feel and behave. Nevertheless, we do not remember all of our experiences, as many of them quickly drop into oblivion, whereas others last as long as life endures^1^. This suggests that some events have a privileged status in producing lasting memories. Specific circumstances may contribute to the selection of occurrences to be retained, thus initiating the processes leading to the establishment of long-term memories. Strong evidence indicates that emotion attains a privileged status in memory^1^, as emotionally arousing events, whether good or bad, are remembered more accurately, clearly and for longer periods of time than neutral ones^2–4^. Long-lasting memories can help an individual deal in the future with similar situations. However, long-lasting memories for aversive experiences have drawn in recent years significant interest, because the disruption of one or more elements of their processing may result in intrusive memories, hence triggering maladaptive responses that can form the basis of various psychiatric disorders, including generalised anxiety, obsessive-compulsive disorders, post-traumatic stress disorder (PTSD) and specific phobias^5^^,^^6^. By understanding the neurobiology of emotional memory, insights can be gained into how it functions normally, and how it may be disrupted in the case of the illnesses cited above, ultimately leading to better treatments for vulnerable patients.

Fear learning is the process of gathering and storing information about the internal and external milieu in situations that evoke fear responses and creates robust memories. These represent long-lasting records of the acquired information that, in turn, modify behaviour when retrieved. The formation of an engram involves acquisition, consolidation and retrieval. It is during encoding, the early phase of consolidation, and retrieval that memory can be more easily modified, either enhanced or impaired^7^. Since memories cannot be directly detected and assayed in experimental animals, their existence is, by necessity, inferred from changes in behaviour following an experience^8^. Nonetheless the preclinical models have strong translational potential. Thus, to study learning and memory, subjects are interrogated by observing their performance in carefully designed behavioural tasks. In the study of fear learning and memory, the most widely used tasks are fear conditioning and inhibitory avoidance^9^. Both paradigms elicit emotional arousal and require basolateral amygdala activation. However, they may engage different circuits and/or cellular machineries at different times during memorisation^9^^,^^10^. Inhibitory avoidance is a procedure that requires a discriminative response, whereas fear conditioning does not require decision-making^11^. Fear conditioning is a form of Pavlovian learning^12^ that involves making an association between aversive stimuli and their consequences. In this paradigm, an initially neutral stimulus, the conditioned stimulus (CS) is paired with an intrinsically aversive stimulus, the unconditioned stimulus (US), which is generally a mild electrical foot-shock. During the training phase, the animal is exposed to a CS paired with the US. The CS is normally represented by the training compartment itself (contextual fear conditioning), or a cue such as a tone or a light that overrides the context^13^. The dependent measure is the time spent freezing, a generalised immobility of all skeletal muscles. After a delay, the context-dependent fear is evaluated by measuring the freezing behaviour of animals placed in the same apparatus in the absence of the US, whereas cued-dependent fear is reflected by measuring freezing in response to tone/light presentation in a different chamber in the absence of the US^13^. The main brain areas involved in contextual and cued fear conditioning include the amygdala, hippocampus, frontal cortex, and cingulate cortex, although with a different temporal profile^14^. The information the animal gathers during the training is the association between the environment or cue and a punishment. At the retention test conducted after a variable time delay, the animal is returned to the previously less-preferred compartment and the latency to enter the non-electrified compartment, is measured. These features have been extensively reviewed by Izquierdo and colleagues^9^a. Animals possess a remarkable ability to associate threatening occurrences with sensory stimuli, e.g. context, smells, and sounds^15^. Such memories persist long after learning^16^, and are crucial for survival, as remembering cues associated with danger allows the animals to select the most appropriate defensive responses^17^. Dreadful memories become labile when retrieved and the processes of extinction ensue. Pavlovian fear conditioning and extinction have become an exemplary model for treating psychiatric disorders characterised by maladaptive elaborations of emotional memories. During memory extinction, the conditioned response gradually diminishes over time as the animal learns to uncouple a response from a stimulus^18^^,^^19^. Contextual fear extinction occurs when a prolonged (minutes) exposure to the context in the absence of additional punishments diminishes or extinguishes the fear response. Hence, extinction is a learning process that does not entail forgetting the initial association. Although we cannot observe a memory trace directly, we infer it from the way it is expressed, and we assume that it is an index of the underlying neural “engram”, the memory trace, supporting a given behaviour. The relationship between certain psychiatric disorders and animal models such as “fear conditioning” are based on behavioural matches, e.g. repetitive actions and stereotyped behaviours^18–20^. Moreover, these experimental paradigms engage brain regions such as the amygdala, hippocampus, infra-limbic and ventromedial prefrontal cortex, that are believed to be crucially involved in emotional processing and to be impaired in the maladaptive responses that are key symptoms of psychiatric disorders such as generalised anxiety, obsessive-compulsive disorders, PTSD and specific phobias^21–24^. As of today, there is no specific pharmacotherapy for these diseases. Brain CAs may represent a previously unexplored mechanism to develop drugs for highly-needed novel treatments of these psychiatric disorders. Our hypothesis stems from the observation that CAs activation ameliorated spatial memory (an emotionally neutral form of memory) in rats^25^. In keeping with these findings, mice genetically deficient of the CA IX isoform performed more poorly in the same task than wild type littermates^26^. More recently, it was reported that administration of the widely used CA inhibitor acetazolamide to CD1 mice reduced CA activity in the brain and caused amnesia in the object recognition (OR) test, whereas treatment with D-phenylalanine (D-Phe) enhanced CA activity and potentiated OR memory as a result of extracellular signal-regulated kinase (ERK) activation^27^. In line with these results, inhibition of CAs also impaired fear memory consolidation in rats through inhibition of ERK phosphorylation^28^. These reports clearly indicate that brain CAs modulation affects the processing of emotional memory. Therefore, a deeper understanding of the role of the carbonic anhydrases in learning and memory has not only a cultural significance but also a translational value. Ligands of CAs, in particular inhibitors, are drugs used in humans as diuretics and for the treatment of glaucoma, epilepsy and obesity^29–32^. Hence understanding the impact of these compounds on learning and memory may help improve their pharmacological profile and unravel unexplored therapeutic applications.

## Carbonic anhydrases

CO_2_ is one of the simplest molecules involved in crucial physiologic processes in all life kingdoms^33–34^. It is generated in most metabolic processes. The carbonic anhydrases (CAs, EC 4.2.1.1) are the metalloenzymes^35–38^, which use CO_2_ as substrates, as they catalyse the interconversion between this molecule and bicarbonate, with the formation of a proton–Equation (1)^35–39^.

(1) $${\text{CO}}_{2}+{\text{ H}}_{2}O{\text{HCO}}_{3}{}^{-}+{\text{ H}}^{+}$$

Although this reaction may occur without a catalyst, at physiological pH values, it is too slow to meet metabolic needs. CO_2_, a poorly water-soluble gas, may damage cellular components (e.g. membranes) if generated in exceedingly high amounts in a cell/tissue, whereas its conversion to water-soluble ions (bicarbonate and protons), interferes with the pH balance of the cell through the generation of the strong acid (H^+^) and a buffering, weak base (HCO_3_^–^)^35–39^. The pH regulation processes as well as homeostasis of bicarbonate and H^+^ ions are tightly controlled processes in all organisms/cells, which make CAs crucial enzymes in many physiological and pathological conditions^35^^,^^36^^,^^40^^,^^41^.

### 2.1. Brain CAs

In humans 15 CAs are expressed, 12 of which are catalytically active: the cytosolic CA I-III, VII and XIII, the membrane-bound CA IV, the mitochondrial CA VA and VB, the secreted (in saliva and tears) CA VI, and the transmembrane CA IX, XII and XIV (the acatalytic forms are CA VIII, X and XI)^35–38^. Some of them (e.g. CA II and IX) are among the most effective catalysts known in nature, possessing a very high turnover number for the hydration of CO_2_ to bicarbonate and protons^35^. The human central nervous system (CNS), as well as the choroid plexus, contains a multitude of CA isoforms, although their functions are not yet completely understood^35^^,^^42^. The highly abundant isoform CA I is expressed in the motor neurons in the human spinal cord. CA II, the physiologically dominant isoform, is expressed in the choroid plexus, oligodendrocytes, myelinated tracts, astrocytes and myelin sheaths in the vertebrates brain^40^^,^^43^. CA III was shown to be present in the choroid plexus, although this isoform has a rather low catalytic activity for the CO_2_ hydration reaction and may possess a different, yet unknown physiological function^40^^,^^43^. The membrane-associated CA IV is located on the luminal surface of cerebral capillaries and associated with the blood-brain barrier, being also concentrated in layers III and VI in the cortex, hippocampus and thalamus of all investigated mammals^43^. The mitochondrial CA VA is also present in the nervous tissues, where the enzyme is expressed in astrocytes and in neurons, being probably involved in biosynthetic processes such as lipogenesis, neoglucogenesis, ureagenesis, etc.^35–39^^,^^44^^,^^45^. Little is known on CA VB in the brain, as no specific studies in this field have been conducted. The expression of CA VII and VIII is rather similar, with relatively high levels being observed throughout the cortex, hippocampus and thalamus, although CA VIII is acatalytic, whereas CA VII shows a good CO_2_ hydrase activity^40^^,^^43^. It should be noted that CA VII is predominantly expressed in the brain, being absent in most other tissues, and it is thus considered a brain-associated CA isoform^35^^,^^40^^,^^43^. CA X was shown to be expressed in the myelin sheath, whereas CA XI is present in the neural cell body and astrocytes in relatively limited regions of the brain (both isoforms are devoid of catalytic activity and their precise physiological functions remain elusive)^43^. CA IX was shown to be overexpressed in many neurologic cancers such as glioma, ependymoma, hemangioblastoma, meningioma and choroid plexus tumours^35^. CA XII is also associated with tumours and has the same expression pattern as CA IX in brain tumours^25^^,^^43^^,^^46^^,^^47^. However, CA XII is also present in normal tissues and a high level of this isoform was reported in the choroid plexus^43^. CA XIII seems not to be present in the brain, whereas CA XIV is expressed in nuclei and nerve tracts associated with pontine, medullary and hippocampal functions^43^. CA XIV was also shown to be located on the plasma membrane of some neurons and on axons of both mouse and human brain^43^.

### CA inhibitors

There are at least four different CA inhibition mechanisms, many of which discovered by our groups^35–40^. The zinc binders possess a zinc-binding group (ZBG), which directly coordinates the metal ion from the enzyme active site.

**Figure 1.**
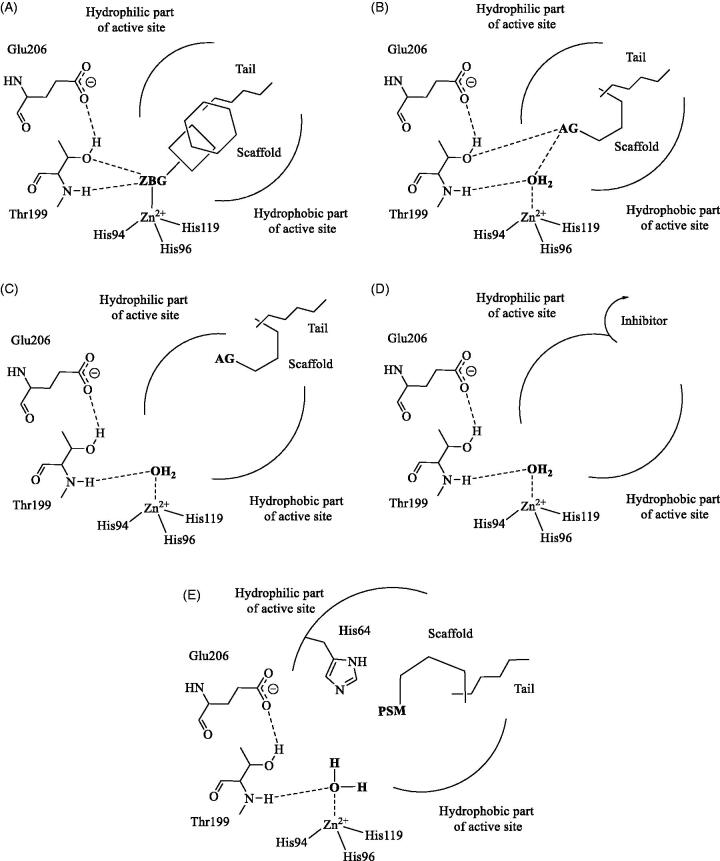
CA inhibition (A–D) and activation (E) mechanisms. The zinc binders incorporate a ZBG (A); the compounds anchoring to the nucleophile an AG that interacts with the zinc-coordinated water (B). The inhibitors occluding the active site entrance (C) also contain AG moieties but bind more externally, whereas the inhibitor binding outside the active site are shown in (D). The activators bind in the middle of the active site and contain a proton shuttle moiety (PSM) of the amine, imidazole or carboxylate type (E). All these modulators incorporate various scaffolds and tails in their molecule.

**Figure 2.**
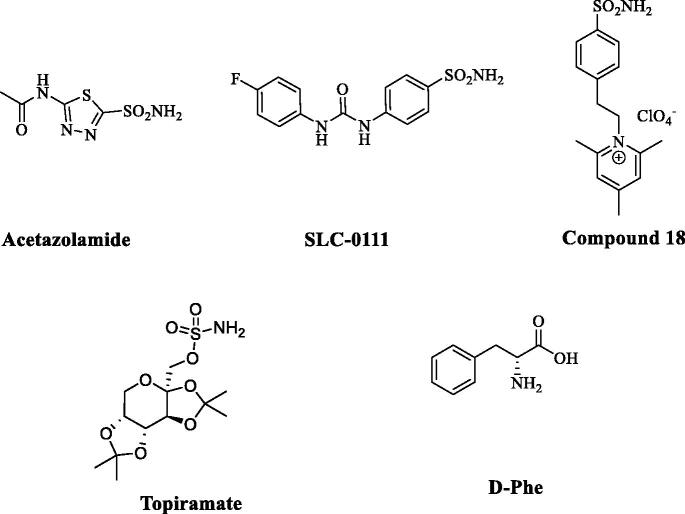
Chemical structures of some CAIs/CAAs. The pan-inhibitor acetazolamide (non-selective inhibitor of all CA isoforms known in humans^35^), the CA IX/XII-selective inhibitor SLC-0111^49^ discovered by our group, in clinical trials as an antitumor agent, the membrane-impermeant positively-charged sulphonamide “Compound 18” (used as negative control in ref ^27^), topiramate, a clinically used antiepileptic agent^48^ together with the non-isoform selective CA activator D-Phe.

The classical inhibitors of the sulphonamide, sulfamate and sulfamide type, but also the recently discovered dithio- and monothiocarbamates, hydroxamates and some carboxylates exert this type of inhibition mechanism (Figure 1(A))^35–37^^,^^48^. Many sulphonamides and some sulfamates have been clinically used for decades as antiglaucoma^29–31^, diuretic^35^, antiepileptic^32^, and antiobesity agents^35–37^^,^^48^, whereas some representatives are in clinical trials as antitumor agents^49^ (among which SLC-0111, discovered in our laboratories, currently in Phase Ib/II clinical trials for the treatment of advanced metastatic tumours, Figure 2 ^49^. The compounds that are anchored to the zinc-coordinated nucleophile possess an anchoring group (AG) by which they interact with the non-protein zinc ligand (Figure 1B). Phenols, polyamines, sulphonates and thioxocoumarins possess this inhibition mechanism^35–37^^,^^48^. Coumarins and structurally related derivatives act as prodrug inhibitors, being hydrolysed by the esterase activity of the enzyme with the generation of 2-hydroxy-cinnamic acid derivatives, which occlude the entrance of the active site (Figure 1(C))^50^. Some carboxylates bind in a hydrophobic pocket outside the active site cavity (Figure 1(D)) and block, by means of a network of hydrogen bonds His64, the proton shuttle moiety of the enzyme, leading to the collapse of the entire catalytic cycle^51^. Of the various classes of compounds exerting such diverse inhibition mechanisms, for the moment only zinc binders of the sulphonamide/sulfamate type show clinical applications as drugs for the pathologies mentioned above, but also for the management of neuropathic pain^41^ and idiopathic intracranial hypertension^40^.

### 2.3. CA activators

The carbonic anhydrase activators (CAAs) are biogenic amines (histamine, serotonin, and catecholamines), amino acids, oligopeptides, or small proteins^38^. The general mechanism of action for the CA activators (CAAs) is shown in Equation (2) ^35^^,^^38^:

(2) $${\text{EZn}}^{2+}-{\text{OH}}_{2}+\;A⇌[{\text{EZn}}^{2+}-{\text{OH}}_{2}-\;A]⇌[{\text{EZn}}^{2+}-{\text{HO}}^{-}-{\;\text{AH}}^{+}]⇌{\text{EZn}}^{2+}-{\text{HO}}^{-}+{\;\text{AH}}^{+}$$

enzyme – activator complexes—

The activator binds within the enzyme active site with the formation of enzyme–activator complexes^38^, in which the activator molecule (which incorporates a proton shuttling moiety, PSM, Figure 1(E)) participates to the rate-determining step of the catalytic cycle, i.e. transfer of protons from the zinc-coordinated water to the external reaction medium, similar to the natural proton shuttle, which is residue His64 (in many CA isoforms)^35–38^^,^^48^. By means of site-directed mutagenesis and extensive kinetic measurements, it has been demonstrated that His64 through its imidazole moiety (with a pKa of around 7), is crucial for proton shuttling and generation of the nucleophilically active species of the enzyme^38^^,^^52^. In such enzyme-activator complexes, the proton transfer is intramolecular, being more efficient compared to the intermolecular transfer to buffer molecules, not bound within the enzyme cavity^38^. Many X-ray crystal structures with amines and amino acid activators were reported, among which histamine, L- and D-His bound to hCA II and hCA I, L- and D-Phe, D-Trp and L-adrenaline bound to hCA II, which confirmed this general CA activation mechanism^35^. The thirteen catalytically active mammalian CAs, (e.g. CA I-VA, VB, VI, VII, IX, XII-XV) were investigated for their interaction with a library of amino acids and amines. The main hurdle with the activators of the amine and amino acid type is their lack of selectivity for the various CA isoforms^38^, which is also the case for many classes of CAIs^35–38^^,^^48^. It is wo rth mentioning that CA targeted drug design studies on CAAs are in their infancy: most of the known activators were identified by screening libraries of amines and amino acids followed eventually by the subsequent derivatization of such compounds (e.g. histamine, histidine, etc.)^38^^,^^53^.

## Carbonic anhydrase and memory

Evidence supporting the hypothesis that CA inhibition impairs memory in humans has come from studies on topiramate, an antiepileptic drug that also inhibits CAs^54^, and on acetazolamide during acute high-altitude exposure^55^. This negative effect on cognition is also supported by preclinical evidence^25^^,^^28^. Findings from studies in healthy humans, neuropsychiatric patients and non-human animals all point to the conclusion that the basic architecture of emotional memory and its mechanisms are conserved across species^5^^,^^56^^,^^57^, thus encouraging translation to the clinic. As a result, much of our knowledge of the neurobiology of emotional memory comes from investigations on fear learning utilising a Pavlovian approach or instrumental association between the environment, or changes in the environment (conditioned stimulus) and a fearsome stimulus^58^ in experimental animals, rodents in particular^9^^,^^20^. Recently, our group showed that CAAs, similarly to the inhibitors, may have pharmacological applications^27^. Administration of CAAs of the amino acid type (D-Phe was used as an activator) leads to enhanced discrimination learning, which is antagonised by the simultaneous administration of an inhibitor of the sulphonamide type (e.g. acetazolamide, a sulphonamide in clinical use since 1954, Figure 2 (ref ^27^). The same study showed that the administration of the D-Phe rapidly activated the extracellular signal-regulated kinase (ERK) pathways, which is involved in critical steps of memory formation, both in the cortex and the hippocampus, two brain areas crucially involved in memory processing^27^. Impairments of fear memory consolidation were also observed in rats^28^. These observations may pave the way for pharmacological applications in fields such as post-traumatic stress disorders (PTSD). Although these studies are rather preliminary, the highly interesting results obtained so far envisage the use of the CA activators for the therapy of memory disorders.

Emotionally arousing experiences, such as fear, create long-term memories that are initially labile, but over time become insensitive to disruption through a process known as consolidation. The ability to form this type of memories is essential for individuals to detect and react to danger. However, disruption in one or more elements of this memory processes results in maladaptive responses such as anxiety and pathological fear that are key symptoms of many psychiatric conditions, including different types of phobia (social phobia, agoraphobia), obsessive-compulsive disorder, generalised anxiety and PTSD. Each phase of memory formation, including extinction, engage discrete brain regions/circuits that store encoded information as a memory trace^59^. For instance, the consolidation phase occurring after the acquisition is crucial to establish the strength/duration of memory^59^. The amygdala is a key region engaged in mediating emotional valence during memory consolidation and extinction^15^^,^^60–63^. The amygdala modulates memory consolidation also through projections to other brain regions, namely the cortex^64^ and the hippocampus^65^^,^^66^. Fear memory formation recruits multiple regions over time, as different brain circuits are recruited at early versus late time points^9^^,^^57^^,^^67^. Studies using post-acquisition intracerebral administration of selective compounds and testing for memory expression or extinction have contributed significantly to identifying crucial and modulatory systems of memory consolidation^68^. Evidence-based on these preclinical studies indicate that neurotransmitters such as acetylcholine, catecholamines, endocannabinoids, GABA, glucocorticoids, glutamate, histamine, serotonin as well as intracellular molecular cascades such as phosphorylated CREB and CaMKII all critically influence memory formation^9^^,^^10^^,^^69^. In this regard, unpublished results from our laboratories demonstrate that acetazolamide, but not a positively-charged, membrane-impermeant pyridinium sulphonamide (compound **18**, 1–(4-sulfamoyl-phenylethyl)-2,4,6-trimethylpyridinium perchlorate, Figure 2), a CAI which does not pass the blood-brain barrier^31^, significantly counteracts the expression of extinction memory. The mechanisms underpinning CAs actions on cognition remain largely unidentified. Modulation of CAs activity alters the buffering capacity, thus influencing intracellular and extracellular pH value, therefore affecting protein NMDA and GABA receptors function [56]. Early studies demonstrated that the associated activation of multi-synaptic inputs on pyramidal neurons in the hippocampal CA1 region transiently transform GABAergic IPSPs to EPSPs; this transformation depends on CA activation as it busts HCO_3_^–^ intracellular concentrations favouring its efflux through the GABA_A_ receptor channel^70^^,^^71^. Therefore, GABA-mediated responses on CA1 pyramidal neurons become excitatory amplifying synaptic weights relevant to a particular memory processing (reviewed in ^72^). Therefore, CAs act as a gate potentiating signal transfer through the neural network. More recently, we demonstrated that treatment with D-Phe augmented significantly ERK 1/2 phosphorylation in hippocampal and cortical homogenates. Such effect was prevented by the co-administration of acetazolamide, whereas the co-administration of compound **18**, a brain-impermeant CAI, did not affect D-Phe-induced effect^27^. These findings were in agreement with previous reports showing that increased ERK 1/2 phosphorylation in the amygdala due to fear conditioning training was also inhibited by acetazolamide treatment^28^. The genomic response activated by ERK pathway is an essential step for the consolidation and persistence of several types of long-term memories^73–75^.

**Figure 3.**
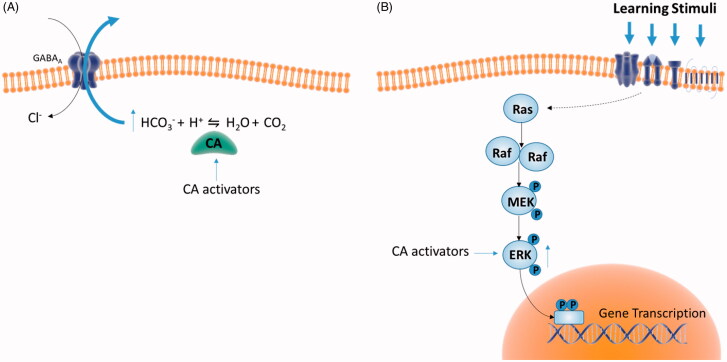
Putative mechanisms underpinning CAs actions on cognition. (A) CA activation transforms GABA-mediated inhibition (Cl^−^ conductance) into excitation due to increased HCO_3_^–^ flux through the GABA_A_ receptor channel. Such synaptic transformation allows GABA-releasing interneurons to act as either excitation filters or amplifiers of the neuronal network^72^. (B) CA activators increase ERK phosphorylation^27^ which in turn regulates the activity of nuclear transcription factors promoting gene transcription, an essential step for consolidation of different learning stimuli^73–75^. The CA isoforms as well as the cellular mechanisms related to CA-induced modulation of ERK activity were not identified yet.

On the other hand, the administration of CAAs, such as D-Phe^53^ leads to enhanced discrimination learning. Therefore, CAAs-induced increased ERK phosphorylation is necessary for memory consolidation, as shown schematically in Figure 3.

## Conclusions and future perspectives

Maladaptive behaviours in response to traumatic events as in PTSD and phobias, contribute significantly to both the personal suffering of patients and the heavy socio-economic burden. The primary treatment of these disorders is exposure therapy, which is based on techniques aimed at recalling and extinguishing the threatening memory. However, extinction does not represent the cancellation of the original memory, but results in a new memory trace that inhibits the expression of the initial memory. Therefore, since the original memory is not cancelled but only inhibited, maladaptive defensive behaviours can reappear over time (spontaneous recovery), with changes in the context (renewal) or in the presence of unexpected stressful situations (reintegration). This potential for the recovery of maladaptive memory highlights the need to discover more persistent and robust techniques to decrease maladaptive behaviours. Treatment strategies could take great advantage from compounds reinforcing the consolidation of extinction. CAs may represent a novel target for these drugs. Preclinical models with strong translational potentials are available, among which the most frequently used is the extinction of Pavlovian fear, a procedure similar to exposure therapy^24^. In this context, more studies are required for a better comprehension of the relation between CAs and brain function as well as to understand, which brain CA isoforms are involved in extinction processes and which brain area might interest this phenomenon. In this context, for example, it is of great interest (i) to design inhibitors and activators (CA modulators), which selectively interact with brain CA isoforms (CA I, II, III, IV, VA, VII, IX, XII and XIV); (ii) to evaluate *in vitro* the ability of such modulators to pass the blood-brain barrier (BBB), as well as their activity against all catalytically active CA isoforms, with a focus on the brain-associated ones; (iii) finally, to evaluate in vivo the effect of the modulators in animal models. Following this strategy, it will be possible to investigate the role played by brain CAs in extinction that refers to the gradual decrease of conditioned responses since the specific neural substrates of extinction are presently poorly understood. This may have a therapeutic relevance since the exposure therapy (the main treatment of psychiatric disorders such as phobia, obsessive-compulsive disorder, generalised anxiety and PTSD) is based on techniques activating extinction, thus taking great advantage from compounds reinforcing extinction memories. The CAs may represent a crucial family of targets for such novel drugs in the therapeutic areas mentioned above, for which there are few effective therapies available at this moment.
